# Is there still a role for bilateral orchidectomy in androgen-deprivation therapy for metastatic prostate cancer?

**DOI:** 10.1080/2090598X.2019.1690270

**Published:** 2019-11-13

**Authors:** Mohamed Adel Atta, Ahmed Elabbady, Wael Sameh, Mohamed Sharafeldeen, Mohamed Elsaqa

**Affiliations:** Department of Urology, Faculty of Medicine, University of Alexandria, Alexandria, Egypt

**Keywords:** Prostate cancer, bilateral orchidectomy, LHRH antagonist, health-related quality of life

## Abstract

**Objective**: To compare bilateral orchidectomy, as the classical ‘gold standard’ androgen-deprivation therapy (ADT), and ADT using a luteinising hormone-releasing hormone (LHRH) antagonist (degarelix) for the treatment of metastatic prostate cancer regarding their short-term biochemical efficacy, testosterone castrate level, tolerability, and effect on health-related quality of life (HRQoL).

**Patients and methods**: A total of 60 patients with newly diagnosed metastatic prostate cancer were managed by either bilateral orchidectomy or degarelix injection as ADT. Both groups were compared according to their prostate-specific antigen (PSA) nadir and testosterone level at the 6-month follow-up. HRQoL was assessed using the European Organisation for Research and Treatment of Cancer (EORTC) Quality of Life Questionnaire-Core 30 (QLQ-C30) after 12 months.

**Results**: Bilateral orchidectomy and degarelix showed comparable results for PSA reduction, but there was a statistically significantly lower castrate level of testosterone in the bilateral orchidectomy group. Using the EROTC QLQC-30, bilateral orchidectomy was associated with better HRQoL, better global health status, and better functional status.

**Conclusion**: Bilateral orchidectomy resulted in lower castrate levels of testosterone, which may be associated with better disease control, together with better HRQoL and general health status compared to LHRH antagonist (degarelix). These results indicate that we should consider revisiting bilateral orchidectomy as a valuable and effective treatment option for ADT.

**Abbreviations**: ADT: androgen-deprivation therapy; EORTC (QLQ-C30): European Organisation for Research and Treatment of Cancer (Quality of Life Questionnaire-Core 30); HRQoL: health-related quality of life

## Introduction

Metastatic prostate cancer has a profound response to androgen deprivation. Since 1941, when Huggins and Hodges proved the favourable effects of androgen deprivation by surgical castration or oestrogen administration on the progression of metastatic prostate cancer, androgen-deprivation therapy (ADT) has been the mainstay for the management of advanced prostate cancer to date [1]. Bilateral orchidectomy is the traditional ‘gold standard’ for androgen deprivation. It is a simple surgical procedure and can even be done under local anaesthesia [2]. Patients with symptomatic metastasis show significant improvement within 24–48 h after bilateral orchidectomy [3]. In patients accepting bilateral orchidectomy, it is considered the most cost-effective form of androgen ablation [4].

For a long time, LHRH agonists have been used as an alternative method of androgen deprivation. LHRH agonists result in suppression of LH from the anterior pituitary gland and consequently, results in an inhibition of testosterone [5,6]. However, LHRH agonists initially activate the receptors, resulting in a surge in LH and testosterone, as well as a delayed reduction in PSA levels for 2–3 weeks before androgen deprivation is achieved [7,8]. The surge can delay the therapeutic benefit and may exacerbate the clinical status by provoking or exacerbating symptoms such as urinary retention, bone pain, and paraplegia due to spinal-cord compression by spinal metastases, this is referred to as the flare-up phenomenon or hormonal surge [7,8].

Novel LHRH antagonists do not induce a testosterone surge but work by immediately by suppressing the release of gonadotrophins and testosterone. Degarelix is a novel LHRH antagonist with weak histamine-releasing properties and a more rapid and profound testosterone suppression compared to trials of other LHRH antagonists [9–11].

Although ADT is effective in the management of prostate cancer, it has various side-effects that may impact on health-related quality of life (HRQoL), especially with long-term ADT. Adverse effects of ADT include hot flushes, sexual dysfunction, fatigue, accelerated osteoporosis, increased body fat, and impaired cognitive functions [12,13]. As prostate cancer is generally a disease of elderly men, many patients present with significant comorbidities that have a detrimental impact on life expectancy. Consequently, the potential benefits of ADT must be weighed against its potential hazards and impact on HRQoL [14,15].

In the present study, we compared bilateral orchidectomy and LHRH antagonist (namely degarelix) as ADT for the treatment of newly diagnosed metastatic prostate cancer regarding efficacy, tolerability, and HRQoL.

## Patients and methods

This prospective study included patients with newly diagnosed metastatic prostate cancer who presented to Alexandria University hospitals between October 2015 and October 2017. All patients had positive bone scans for metastasis. Patients were counselled about both methods of androgen deprivation and they were assigned to one of the two groups according to their preferences:

- Group I: Patients treated by bilateral orchidectomy.
- Group II: Patients treated by subcutaneous degarelix (Firmagon^TM^; Ferring Pharmaceuticals, Saint-Prex, Switzerland) injection. Loading dose of 240 mg and maintenance dose of 80 mg monthly injections were given.

All patients were subjected to the following:

- Baseline detailed assessment.
- Follow-up hormonal assessment of serum PSA and serum testosterone at 6 months after the start of hormonal therapy.
- Assessment of HRQoL using the validated Arabic version [16,17] of the European Organisation for Research and Treatment of Cancer (EORTC) Quality of Life Questionnaire-Core 30 (QLQ-C30) at the 12-month follow-up [18].

The QLQ-C30 includes 30 questions that assess both physical and psychological aspects of the patient’s health. It assesses the ability to perform usual physical and social activities, as well as general physical complaints such as fatigue, nausea, vomiting, constipation, and dyspnoea. From the questionnaire, three main items can be evaluated; the functional scale, symptom scale, and global health scale. Each scale is measured by a score ranging from zero to 100.

For statistical analysis, the chi-square, Fisher’s exact or Monte Carlo correction tests were used to determine a significant relationship between nominal/categorical variables, whilst the Student’s *t*-test or Mann–Whitney test were used for ordinal/continuous variables. The *P* value for statistical significance was set at *P* < 0.05.

## Results

The study included 60 consecutive patients; 33 in Group I and 27 in Group II. All the patients in the degarelix arm continued the treatment to the 12-month follow-up. The mean (SD) age was 67 (7) years in both groups. The mean (SD) initial PSA level was 240.8 (43) ng/mL in Group I and 306 (63) ng/mL in Group II. There was no significant statistical difference between the groups for age and initial presenting serum PSA level. Using the new International Society of Urological Pathology (ISUP) Gleason groups [19], there was a tendency towards more aggressive Gleason groups in Group I, with 18 patients in Group I with Gleason Group 4 or 5, whereas in Group II there were only eight patients with high grades (Table 1).

**Table 1.** Initial presenting data of both groups.

Variable	Group I (bilateral orchidectomy)	Group II (degarelix)	*P*
**Number of patients**	33	27	
**Age, years, mean (SD)**	67.73 (7.24)	67.80 (7.73)	0.973*
**Gleason Groups, *n* (%)**			0.027**
Group 1	6 (18.2)	2 (7.4)	
Group 2	3 (9.1)	2 (7.4)	
Group 3	6 (18.2)	15 (55.6)	
Group 4	10 (30.3)	2 (7.4)	
Group 5	8 (24.2)	6 (22.2)	
**Serum PSA level (ng/mL), *n* (%)**			0.912**
<20	4 (12.1)	4 (14.8)	
20–50	5 (15.2)	3 (11.1)	
>50	24 (72.7)	20 (74.1)	
**Symptoms at presentation, *n* (%)**			
LUTS	33 (100)	25 (92.5)	0.492***
Bone pain	21 (63.6)	15 (55.6)	0.791
Obstructive uropathy	6 (18.2)	0	0.024

*Chi-square test, **Monte Carlo correlation, ***Fisher’s exact correlation.

Most of the patients presented with LUTS or bone pain. None of the degarelix-group patients (Group II) presented with obstructive uropathy, whereas six of the 33 patients (18.2%) in the bilateral orchidectomy group (Group I) presented with obstructive uropathy and were managed by nephrostomy tube insertion. The patients who presented with obstructive uropathy in Group I were aged >60 years (range 60–72 years) and Gleason Group 4 or 5 (Table 1).

### Serum PSA and serum testosterone levels

The presenting and follow-up PSA and percentage of PSA reduction between presentation and post-treatment follow-up were compared in both groups as shown in Table 2. After 6 months of treatment, in Group I the follow-up PSA level varied between 0.1 and 34 ng/mL, with a mean (SD) PSA level of 9.37 (10.04) ng/mL; while in Group II the follow-up PSA level varied between 0.02 and 34.5 ng/mL, with a mean (SD) PSA level of 7.55 (11.79) ng/mL. The initial and follow-up PSA levels were not significantly different in both groups.

**Table 2.** Comparison between the two studied groups according to serum PSA and testosterone castrate levels.

Variable	Group I (bilateral orchidectomy)	Group II (degarelix)	P*
Initial PSA level, ng/mL, mean (SD; range)	240.89 (433.19; 1–1935)	305.47 (632.81; 9–2161)	0.767
Follow-up PSA level (at 6 months), ng/mL, mean (SD; range)	9.37 (10.04; 0.1–34.0)	7.55 (11.79; 0.02–34.5)	0.108
% PSA reduction, mean (SD)	91.92 (12.31)	93.14 (10.22)	0.676
Serum testosterone castrate level, ng/mL, mean (SD; range)	0.178 (0.197; 0.02–0.7)	0.297 (0.125; 0.09–0.49)	0.001

*Mann–Whitney test.

The mean (SD) percentage PSA reduction in Group I was 91.9 (12.3)% compared to the presenting levels and in Group II was 93.1 (10.2)%. There was no statistically significant difference between the groups. The follow-up serum testosterone level in Group I varied from 0.002 to 0.7 ng/mL, with a mean (SD) testosterone level of 0.178 (0.197) ng/mL; while in Group II the testosterone level varied from 0.09 to 0.49 ng/mL, with a mean (SD) testosterone level of 0.297 (0.125) ng/mL. There was a statistically significantly lower serum testosterone level in Group I, as shown in Table 2.

### HRQoL assessment with the QLQ-C30

**Figure 1.**
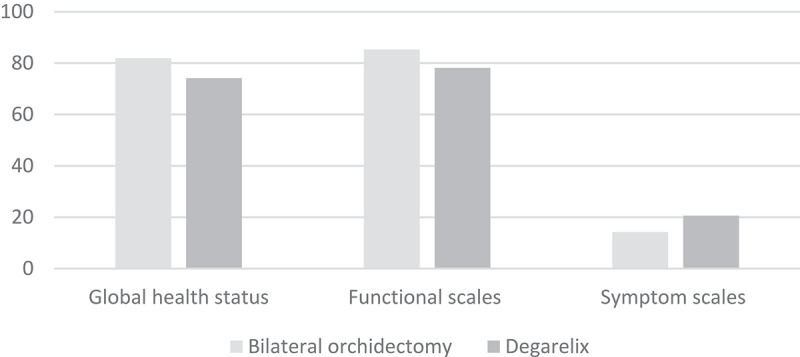
Comparison between HRQoL scales in both groups.

The Arabic version of the EORTC QLQ-C30 questionnaire completed by the patients at the 12-month follow-up was analysed and the scoring system was applied to evaluate the functional scale, symptom scale and global health scale. The mean (SD) global health status was 81.94 (10.51)% in Group I and 74.17 (11.23)% in Group II (*P* < 0.008). The mean (SD) functional scale score was 85.26 (7.66)% in Group I and 78.15 (6.23)% in Group II (*P* < 0.001). The mean (SD) symptom scale score was 14.19 (6.31)% in Group I and 20.60 (6.26)% in Group II (*P* < 0.001). Thus, Group I had a significantly better global health status and functional scale score, and lower symptom scale score than Group II (Figure 1).

## Discussion

In our present study, bilateral orchidectomy and degarelix had comparable results for reducing the PSA level at 6 months of follow-up; however, the castrate level of testosterone was significantly lower with bilateral orchidectomy. Although the castrate level of testosterone is considered by the regulatory authorities, the threshold that has been used in all clinical trials is still 50 ng/dL (1.7 nmol/L). Oefelein et al. [3] reported that the median testosterone value after bilateral orchidectomy was 15 ng/dL and they considered a castrate level as <20 ng/dL (1 nmol/L). Other studies have highlighted the possibility that lower testosterone levels may be associated with improved outcomes, including increased time to the development of castrate-resistant disease and overall survival [20,21]. In a series of 162 men with metastatic prostate cancer treated with LHRH agonist, Perachino et al. [20] reported that the prognosis was found to be related to 6-month testosterone levels, with longer survival with lower testosterone levels. Similarly, Morote et al. [21] strongly suggested that patients experiencing a breakthrough testosterone during LHRH agonist therapy have a reduced progression-free survival rate compared with those who did not experience testosterone breakthroughs.

Our present study is the first to compare degarelix to bilateral orchidectomy. Multiple studies have evaluated the efficacy and tolerability of degarelix in comparison to LHRH agonists. Klotz et al. [22] analysed the pooled data of five randomised comparative clinical trials of degarelix vs LHRH agonists. The pooled data showed that PSA progression-free and overall survival were improved in the degarelix group. Also, degarelix was associated with fewer joint-related signs and symptoms, musculoskeletal events, and urinary tract events.

Due to the concern about the adverse effects of hormonal therapy, HRQoL and global health status in patients receiving hormonal therapy for prostate cancer has been a focus of many studies. Two recent studies have compared the incidence of adverse events between surgical castration and LHRH agonists [23,24]. The studies showed that bilateral orchidectomy was associated with a reduced risk of adverse events, especially skeletal-related events, peripheral arterial disease, cardiac-related complications, and diabetes mellitus.

With the use of the EORTC QLQ-C30, the general HRQoL was assessed in both of the present groups. The results of our present study have shown that bilateral orchidectomy was associated with better HRQoL and global health status with a lesser symptoms score than patients who used degarelix. The reason for a better HRQoL in the case of bilateral orchidectomy is uncertain, but this may be attributed to the lower testosterone castration level, which could reflect better disease control. Although concerns about psychological insult with surgical castration is always raised, patients that choose bilateral orchidectomy may experience less worry and concern about cancer control than patients receiving monthly degarelix injections.

The limitations of the present study include: the small number of patients, the non-randomised nature of the study, the lack of a pre-treatment HRQoL assessment, and the short-term follow-up. Further studies with long-term follow-up and a larger number of cases may be needed.

## Conclusion

Although degarelix has shown considerable effectiveness and safety for the management of prostate cancer, bilateral orchidectomy was associated with lower testosterone levels, which may reflect better disease control, and better HRQoL and general health status. These results indicate that we should consider revisiting bilateral orchidectomy as a valuable and effective treatment option for ADT.
